# Evaluation of a Machine Learning Model Based on Pretreatment Symptoms and Electroencephalographic Features to Predict Outcomes of Antidepressant Treatment in Adults With Depression: A Prespecified Secondary Analysis of a Randomized Clinical Trial

**DOI:** 10.1001/jamanetworkopen.2020.6653

**Published:** 2020-06-22

**Authors:** Pranav Rajpurkar, Jingbo Yang, Nathan Dass, Vinjai Vale, Arielle S. Keller, Jeremy Irvin, Zachary Taylor, Sanjay Basu, Andrew Ng, Leanne M. Williams

**Affiliations:** 1 Department of Computer Science, Stanford University, Stanford, California; 2 Stanford Center for Precision Mental Health and Wellness, Department of Psychiatry and Behavioral Sciences, Stanford University, Stanford, California; 3 Center for Primary Care, Harvard Medical School, Boston, Massachusetts; 4 Research and Analytics, Collective Health, San Francisco, California; 5 Division of Primary Care and Public Health, Imperial College London School of Public Health, London, United Kingdom

## Abstract

**Importance:**

Despite the high prevalence and potential outcomes of major depressive disorder, whether and how patients will respond to antidepressant medications is not easily predicted.

**Objective:**

To identify the extent to which a machine learning approach, using gradient-boosted decision trees, can predict acute improvement for individual depressive symptoms with antidepressants based on pretreatment symptom scores and electroencephalographic (EEG) measures.

**Design, Setting, and Participants:**

This prognostic study analyzed data collected as part of the International Study to Predict Optimized Treatment in Depression, a randomized, prospective open-label trial to identify clinically useful predictors and moderators of response to commonly used first-line antidepressant medications. Data collection was conducted at 20 sites spanning 5 countries and including 518 adult outpatients (18-65 years of age) from primary care or specialty care practices who received a diagnosis of current major depressive disorder between December 1, 2008, and September 30, 2013. Patients were antidepressant medication naive or willing to undergo a 1-week washout period of any nonprotocol antidepressant medication. Statistical analysis was conducted from January 5 to June 30, 2019.

**Exposures:**

Participants with major depressive disorder were randomized in a 1:1:1 ratio to undergo 8 weeks of treatment with escitalopram oxalate (n = 162), sertraline hydrochloride (n = 176), or extended-release venlafaxine hydrochloride (n = 180).

**Main Outcomes and Measures:**

The primary objective was to predict improvement in individual symptoms, defined as the difference in score for each of the symptoms on the 21-item Hamilton Rating Scale for Depression from baseline to week 8, evaluated using the C index.

**Results:**

The resulting data set contained 518 patients (274 women; mean [SD] age, 39.0 [12.6] years; mean [SD] 21-item Hamilton Rating Scale for Depression score improvement, 13.0 [7.0]). With the use of 5-fold cross-validation for evaluation, the machine learning model achieved C index scores of 0.8 or higher on 12 of 21 clinician-rated symptoms, with the highest C index score of 0.963 (95% CI, 0.939-1.000) for loss of insight. The importance of any single EEG feature was higher than 5% for prediction of 7 symptoms, with the most important EEG features being the absolute delta band power at the occipital electrode sites (O1, 18.8%; Oz, 6.7%) for loss of insight. Over and above the use of baseline symptom scores alone, the use of both EEG and baseline symptom features was associated with a significant increase in the C index for improvement in 4 symptoms: loss of insight (C index increase, 0.012 [95% CI, 0.001-0.020]), energy loss (C index increase, 0.035 [95% CI, 0.011-0.059]), appetite changes (C index increase, 0.017 [95% CI, 0.003-0.030]), and psychomotor retardation (C index increase, 0.020 [95% CI, 0.008-0.032]).

**Conclusions and Relevance:**

This study suggests that machine learning may be used to identify independent associations of symptoms and EEG features to predict antidepressant-associated improvements in specific symptoms of depression. The approach should next be prospectively validated in clinical trials and settings.

**Trial Registration:**

ClinicalTrials.gov Identifier: NCT00693849

## Key Points

**Question:**

Can machine learning models predict improvement of various depressive symptoms with antidepressant treatment based on pretreatment symptom scores and electroencephalographic measures?

**Findings:**

In this prognostic study, using the machine learning approach of gradient-boosted decision trees, the ElecTreeScore algorithm could reliably distinguish the patients who responded to treatment from those who did not based on various depressive symptoms using pretreatment symptom scores and electroencephalographic features (using the cross-validation approach on 518 patients).

**Meaning:**

Machine learning approaches that include pretreatment symptom scores and electroencephalographic features may help predict which depressive symptoms will improve with antidepressants.

## Teaser

This prognostic study examines the extent to which a machine learning approach, using gradient-boosted decision trees, can predict acute improvement in individual depressive symptoms with antidepressants based on pretreatment symptom scores and electroencephalographic measures.

## Introduction

Major depressive disorder (MDD) is the second leading cause of years lived with disability worldwide, affecting 16 million adults in the United States each year.^1^ Typically less than 50% of patients with MDD respond (≥50% reduction in depressive symptoms) to their initial antidepressant medication and even fewer achieve remission (symptoms return to the healthy range).^2^ Clinicians must decide for each patient whether antidepressant treatment is likely to increase the chances of response and ideally remission, weighing the benefits against the undesirable outcomes, including adverse effect burden.^3^

The Hamilton Rating Scale for Depression (HRSD) is a widely used test to quantify the severity of illness in patients with a diagnosis of depression.^4,5^ The HRSD consists of 17 symptoms of depression—including loss of weight, thoughts of suicide, and feelings of guilt—which are rated on either a 3-point or 5-point scale, and 4 additional symptoms that are used to subtype depression but not to assess its severity. Most studies of depression sum all of the 17 symptoms to a single score for assessing severity of depression, treating depression as a single, unidimensional, condition.^6^

However, there is evidence that depression is not a single condition but a widely heterogeneous set of conditions.^7,8,9^ Two individuals with equal HRSD total scores may have very different clinical conditions^10^; specific depressive symptoms such as sad mood, insomnia, and suicidal ideation may be understood as distinct phenomena that differ from each other in important dimensions. Electroencephalographic (EEG) measures have shown significant potential as objective biomarkers for MDD, with accumulating evidence that pretreatment quantitative EEG measures may be useful for prediction of antidepressant response and remission for patients with MDD.^11,12,13,14,15^ However, we lack an understanding of whether EEG biomarkers predict improvement in specific clinical symptoms as well as robust toolkits to use in making such predictions.^10,16,17^

Understanding the association between EEG-recorded neural activity and response to antidepressant medication for patients with MDD has long been a topic of inquiry. Prior studies have highlighted the relevance of particular EEG frequency bands in antidepressant response. For example, patients who did not respond to antidepressants have been characterized by relatively elevated theta power at rest,^18,19^ although the reverse outcome of relative reduced theta has also been observed.^20^ Using source localization, theta activity relevant to predicting response among those taking fluoxetine hydrochloride or venlafaxine hydrochloride has been localized to the rostral anterior cingulate and medial orbitofrontal regions.^14^ A distinct profile of alpha power has been associated with antidepressant response. For example, response (rather than nonresponse) to antidepressants has been associated with elevated alpha source density.^11^ Other lines of investigation have examined metrics for quantifying alpha asymmetry. Although there is evidence that relatively greater right-sided alpha distinguishes patients who responded to antidepressants from those who did not,^21^ other studies observe such an alpha asymmetry effect only in women with depression.^22^

Although, to our knowledge, there is little work using EEG biomarkers to probe drug-specific antidepressant effects, one analysis from the International Study to Predict Optimized Treatment in Depression (iSPOT-D) indicated that abnormalities in EEG peak alpha may be alleviated by sertraline hydrochloride in particular.^23^ By contrast, alpha peak frequency may predict a poorer response among patients taking escitalopram oxlate and extended-release venlafaxine hydrochloride.^24^ Another study using data gathered by CAN-BIND (Canadian Biomarker Integration Network for Depression) found that the patients who responded to escitalopram were identified by elevated absolute alpha and relative delta power in the left hemisphere, whereas the patients who did not respond to escitalopram showed the opposite.^25^ Machine learning methods have been used to identify EEG features predictive of symptom response to other psychoactive drugs, such as clozapine.^26^ These studies show that EEG features are not only useful for predicting improvement in general but may also be useful differential predictors of improvement.

In this study, we developed the ElecTreeScore algorithm, a machine learning model to predict the treatment response of antidepressant medications for each symptom of the HRSD based on pretreatment EEG in addition to symptom severity. We developed the ElecTreeScore using data from iSPOT-D,^27^ which has a sufficiently large sample to obtain reliable associations between EEG markers and individual symptoms, and validated the predictive performance of the machine learning model on a holdout test set. We investigated the most important HRSD and EEG features for the prediction and the outcome of depression using the HRSD and EEG features in combination vs using either alone. This approach afforded the opportunity to identify the association of baseline symptoms and EEG features and to evaluate the extent to which EEG features are associated with depression over and above symptom severity. Drawing on prior findings from the application of EEG in characterizing antidepressant response, our study investigated whether a machine learning approach, using gradient-boosted decision trees (GBDTs), could accurately predict acute improvement in individual depressive symptoms with antidepressants based on pretreatment symptom scores and EEG.

## Methods

The study was approved by each site’s governing institutional review board (Stanford University; St Louis University; The Ohio State University; University of Virginia; Shanti Clinical Trials; Center for Healing the Human Spirit; Skyland Behavioral Health Associates; NeuroDevelopment Center, Brown University; Brain Resource Center, Columbia University; University of Sydney, Westmead Hospital; Monash University, Alfred Hospital; Swinburne University; Flinders University; Auckland University; Kings College Institute of Psychiatry; Brainclinics Diagnostics & Treatment, Nijmegen, University; and Brain Health, University of Wittswatersrand) and was carried out in accordance with the Declaration of Helsinki.^28^ Institutional review board approval was obtained prior to patient enrollment at each participating site. All participants provided written informed consent after all of the study procedures and potential risks and benefits had been fully explained. The Transparent Reporting of a Multivariable Prediction Model for Individual Prognosis or Diagnosis (TRIPOD) reporting guideline was used for the reporting of this study.

### Data Set

The data set used in this study was collected as part of iSPOT-D, an international multicenter, randomized, prospective open-label trial aimed at identifying clinically useful predictors and moderators of response to 3 of the most commonly used first-line antidepressant medications. As previously outlined, iSPOT-D included 1008 adults (aged 18-65 years) enrolled between December 1, 2008, and September 30, 2013, with a diagnosis of current nonpsychotic MDD. Participants were enrolled when they were unmedicated (either antidepressant naive or after a washout period of ≥5 half-lives of each drug) and subsequently randomized in a 1:1:1 ratio to 8 weeks of treatment with escitalopram (n = 162), sertraline (n = 176), or extended-release venlafaxine (n = 180).^27^ Because a pragmatic design was used to deliberately mimic real-world practice in which the goal is to select among active treatments, no placebo control was included.

At the baseline and week 8 clinic visits, the severity of the participant’s depressive symptoms was rated on the 21-symptom HRSD (HRSD-21). Study clinical personnel made the ratings based on the participant’s reported information during a semistructured interview. Ten of the HRSD-21 symptoms are rated on a 5-point scale (0 = absent; 1 = doubtful or mild; 2 = mild to moderate; 3 = moderate to severe; and 4 = very severe), while the other 11 symptoms are rated on a 3-point scale (0 = absent; 1 = doubtful or mild; and 2 = clearly present).

In addition, electrophysiological measures were also acquired; resting-state EEG was recorded for 2 minutes while participants were relaxed with eyes closed and eyes open. Electroencephalograms were continuously recorded from 26 sites in 5 regions (frontal, temporal, central, parietal, and occipital) with a NuAmps system (Compumedics) and QuickCap (Compumedics). For each site, we computed absolute and relative band powers for the delta, theta, alpha, beta, and gamma bands.

**Figure 1.**
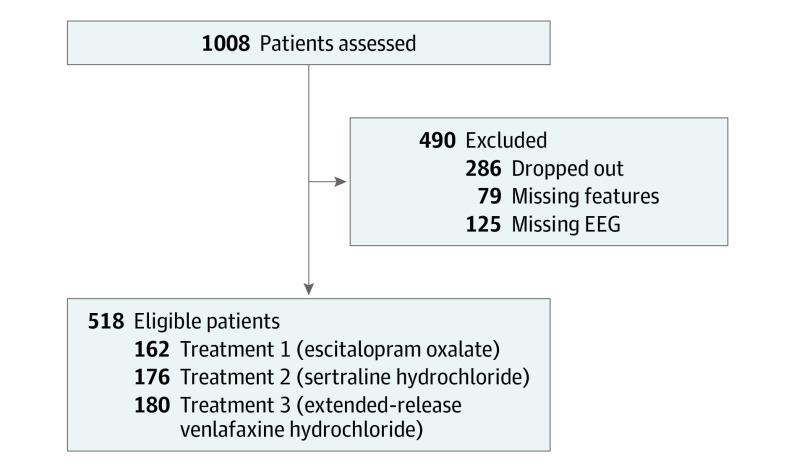
Patient Flow Diagram EEG indicates electroencephalogram.

The data available for the study were from the first 1008 participants with MDD, of whom we excluded those who dropped out (n = 286), those with missing EEGs (n = 125), and those with missing features (n = 79). Previously published work using the iSPOT-D data set has shown that there are no significant differences in attrition across treatment groups^29^ and no significant differences in baseline HRSD scores between those who completed the study and those who dropped out.^30^ The flow of patients for the resulting data set (n = 518) is summarized in Figure 1. The statistics for the HRSD score at baseline and after treatment are shown in eTable 1 of the Supplement. The iSPOT-D study was approved by the institutional review boards at all of the participating sites, and the associated trial was registered with ClinicalTrials.gov (NCT00693849).

### Symptom Improvement Prediction

Our primary objective was to predict improvement in individual symptoms, defined as the difference in score for each of the symptoms on the HRSD-21 report from the baseline visit to the week 8 clinical visit using pretreatment EEG features. We first extracted electrophysiological features from the raw EEGs recorded at the baseline visit and then developed a machine learning approach for the prediction task.

#### Extracting EEG Features

Pretreatment EEG recordings at the baseline visit were processed to generate EEG features. Data on the power of the EEG signals in each frequency range at each electrode site were extracted using the Welch method for spectral density estimation. Specifically, the Welch method was carried out by dividing the EEG signal into successive overlapping windows forming the periodogram for each block and then averaging; the Hanning window was chosen to reduce the side-lobe level in the spectral density estimate, with an overlap of 50% to tradeoff between frequency resolution and smoothness. At each electrode, the absolute power and the relative power were computed using the Simpson rule for the frequency ranges of delta (0.5-4 Hz), theta (4-8 Hz), alpha (8-12 Hz), beta (12-30 Hz), and gamma (30-100 Hz). Two additional features were computed: a frontal alpha asymmetry feature by subtracting alpha power for a left scalp site (F3) from the homologous right site (F4) and a beta-alpha ratio feature by taking the ratio of the beta features at each of the sites with the corresponding alpha features. Furthermore, power features were optionally filtered to only include occipital sites (O1, Oz, and O2) and/or frontal sites (F7, F3, Fz, F4, and F8).

#### ElecTreeScore Algorithm

We developed ElecTreeScore, a machine learning model using GBDTs for the task of predicting improvement in individual symptoms using pretreatment EEG and baseline HRSD scores. Gradient-boosted decision trees are a type of machine learning model that can capture nonlinear associations in data that traditional linear models are unable to capture and can handle mixes of categorical and continuous covariates.^31^ The training procedure for GBDTs involves the construction of an ensemble of decision trees such that each tree learns from the errors of the prior tree to iteratively improve predictions.^32^ Concretely, with each iteration, a new tree is constructed by sampling from the data and first identifying which variable most effectively divides the members into groups with low within-group variation in symptom improvement and high between-group variation in symptom improvement; then, the variable selection process is repeated to further divide each resulting subset of the data, producing a series of branches in the decision tree. The next tree is fit using the same process on the residuals of the previous learner. The implementation details for the model are detailed in the eAppendix in the Supplement.

We trained GBDTs for each of the 21 HRSD categories across several possible combinations of both input features and parameters for the model. Models were trained on valid combinations of EEG bands, relative and absolute power for frequency bands, electrode site–specific features, and asymmetry features. The combination process first chooses whether to use relative or absolute power, then iterates over combinations of EEG bands, including alpha, beta, delta, theta, and gamma bands (1 possible selection is choosing only alpha and beta bands). Finally, the process iterates over regions where EEG bands are obtained, namely the frontal and occipital regions. After the EEG feature selection process, a list of input features, such as “Fz alpha absolute,” were chosen by the algorithm. We use terms such as “Fz alpha absolute” as abbreviations to communicate which regions, bands, and power metric (absolute or relative) are reported in the results. Coupled with the input feature search is a grid search across GBDTs parameters, including the number of estimators, the maximum depth of each tree, and the number of leaves. The possible combinations of both input features and parameters for the models, as well as the details for the stratified k-fold validation, are detailed in the eAppendix in the Supplement.

### Statistical Analysis

Statistical analysis was conducted from January 5 to June 30, 2019. We evaluated the performance of the improvement prediction models on their discriminative ability. Discrimination measures a predictor’s ability to separate patients with different responses. The C index, a widely applicable measure of predictive discrimination and a generalization of the area under the receiver operating characteristic curve statistic, is defined as the proportion of all usable patient pairs in which the predictions and outcomes are concordant.^33^ Concretely, the interpretation of the C index is the probability that the algorithm will correctly identify, given 2 random patients with different improvement levels, which patient showed greater improvement. We also reported model goodness of fit using the coefficient of determination (*R*^2^) and the mean absolute error using output after model calibration. The calibration is computed between training outputs of GBDT and the corresponding ground truth value. A linear regression with square regularization loss (ie, least absolute shrinkage and selection operator) using a regularization coefficient of 0.01 was chosen to be the calibration model. We have also reported model calibration using regression slope and intercept. We computed 95% CIs for these metrics using the nonparametric bootstrap with 1000 bootstrap replicates.

The model was trained and validated using k-fold–stratified cross-validation with k set to 5. In this procedure, the data set was randomly partitioned into 5 equally sized subsamples (with no patient overlap) consisting of an approximately equal percentage of each class. In the cross-validation procedure, of the k subsamples, a single subsample was retained as the validation data for testing the model, and the remaining k − 1 subsamples were used as training data. The cross-validation process was then repeated k times, with each of the k subsamples used exactly once as the validation data. The predictions on the k subsamples were then pooled, and the C index was computed; we assessed the variability in our estimates of the C index by using the nonparametric bootstrap with 1000 bootstrap replicates on the pooled cohort.

#### Feature Importances

We used SHAP (Shapley Additive Explanations) to quantify the effect of each feature on the models.^34^ Shapley values explain a prediction by allocating credit among the various input features (such as “Fz alpha absolute,” interpreted as “absolute alpha bandpower at the medial frontal [Fz] site”); feature credit is calculated as the change in the expected value of the model’s prediction of improvement for a symptom when a feature is observed vs unknown. To uncover clinically important EEG features that were globally predictive of the improvement for each of the individual symptoms on the HRSD, we aggregated the Shapley values for features on individual predictions and reported the top features per model along with their averaged Shapley contributions as a percentage of the associations of all the features.

#### Using Both EEG and Baseline Symptoms vs Using Baseline Symptoms Alone

We assessed whether the combination of baseline symptom scores and EEG features provide additional predictive value for symptom improvement compared with the baseline symptom scores alone. Thus, for each symptom, we trained additional models that used only the baseline symptom scores as input. We computed the increase in the C index of the default (EEG + HRSD) models compared with models that contained only baseline symptom scores.

#### Incorporation of Treatment Group

As an exploratory analysis, we assessed whether the incorporation of the treatment group would increase the performance of the models in the prediction of symptom improvement. For each item, we retrained the model with inclusion of 3 binary features indicating the presence of each treatment, using the same EEG input features as in the model without the treatment group, and tuning the model across the same grid search parameters. We computed the difference in the C index of the models with and without the additional treatment features.

Our implementation used Python, version 3.6.8 (Python Software Foundation), using the LightGBM, version 2.2.3 (Microsoft) implementation for GBDTs; scikit-learn, version 0.20.2 (scikit-learn developers) for stratified k-fold cross-validation and grid search; and SHAP, version 0.29.1^34^ for computing feature importances.

## Results

The resulting data set contained 518 patients (274 women; mean [SD] age, 39.0 [12.6] years; mean [SD] HDRS-21 score improvement, 13.0 [7.0]). Table 1 details the mean (SD) values for the improvement for the 21 symptoms.

**Table 1.** Distribution of the Improvement Outcome (Symptom Score at Week 8 Minus Symptom Score at Baseline) on Each of 21 Symptoms on the HRSD-21 Report in the Data Set Set

Item	Symptom	Magnitude of treatment-related symptom improvement, mean (SD)^a^
1	Depressed mood	−1.53 (0.95)
2	Self-critical	−1.12 (1.03)
3	Suicidal thoughts	−0.44 (0.71)
4	Trouble sleeping	−0.67 (0.87)
5	Nighttime awakening	−0.63 (0.90)
6	Waking early	−0.58 (0.91)
7	Loss of interest	−1.57 (1.11)
8	Psychomotor retardation	−0.62 (0.75)
9	Agitation	−0.69 (0.91)
10	Worrying	−1.14 (0.99)
11	Physical anxiety	−0.72 (0.92)
12	Appetite changes	−0.46 (0.79)
13	Energy loss	−0.89 (0.75)
14	Libido loss	−0.49 (0.86)
15	Health preoccupation	−0.19 (0.58)
16	Weight loss	−0.31 (0.71)
17	Loss of insight	−0.08 (0.32)
18	Diurnal variation	−0.38 (0.82)
19	Unreality and nihilism	−0.26 (0.70)
20	Paranoia	−0.16 (0.52)
21	Obsessive thoughts	−0.09 (0.43)

Abbreviation: HRSD-21, 21-Item Hamilton Rating Scale for Depression.

^a^ Negative values for mean magnitude change are indicative of improvement in symptoms.

### Machine Learning Evaluation

**Figure 2.**
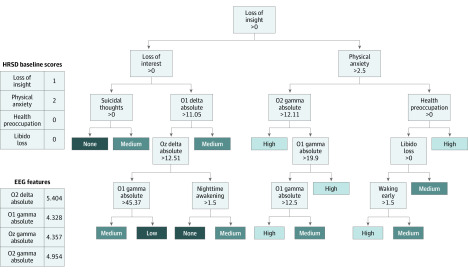
The ElecTreeScore Algorithm Applied to a Sample Patient in the Data Set to Predict Level of Improvement of the Loss of Insight Depressive Symptom Left, the electroencephalographic (EEG) features and Hamilton Rating Scale for Depression (HRSD) baseline features for the test patient at baseline. Four of the HRSD features and 4 of the EEG features are depicted as examples. Right, one of the decision trees used by ElecTreeScore to make its prediction. The light gray boxes correspond to decision points where left branches are followed when the feature value is smaller than the decision boundary, while right branches are followed when the feature value is larger than the decision boundary. The other boxes that are different, darker shades of gray correspond to the level of treatment response predicted by the model. The categories of “none,” “low,” “medium,” and “high” are used for the purposes of visualizing and communicating the results, without losing the essence of the statistical findings.

The machine learning model achieved C index scores, indicative of discriminative performance, of 0.8 or higher on 12 of 21 clinician-rated symptoms. The highest C index scores for prediction of improvement were for the following symptoms: loss of insight (C index, 0.963 [95% CI 0.939-1.000]), unreality and nihilism (C index, 0.951 [95% CI, 0.932-0.976]), and weight loss (C index, 0.923 [95% CI, 0.896-0.953]) (Table 2). The lowest C index scores were for the following symptoms: depressed mood (C index, 0.662 [95% CI, 0.633-0.700]), energy loss (C index, 0.676 [95% CI, 0.637-0.713]), and loss of interest (C index, 0.679 [95% CI, 0.647-0.710]). The performances of the machine learning model on each symptom are detailed in Table 2. An example of the machine learning model applied to a sample patient in the data set is illustrated in Figure 2.

**Table 2.** Performance of Machine Learning Model on Predicting the Improvement for Each Symptom of the HRSD-21 Depression Assessment Scale Using Pretreatment EEG Features and Baseline HRSD-21 Scores

Symptom and most important features^a^	Contribution, %	C index (95% CI)
Waking early		
Waking early	64.3	0.835 (0.808-0.858)
Self-critical	8.8	
Nighttime awakening	8.5	
Physical anxiety		
Physical anxiety	62.2	0.805 (0.772-0.83)
Paranoia	3.6	
O1 alpha absolute	3.0	
Trouble sleeping		
Trouble sleeping	57.3	0.773 (0.741-0.801)
T7-T3 alpha absolute ratio	6.7	
T7-T3 beta absolute ratio	4.4	
Self-critical		
Self-critical	52.8	0.743 (0.714-0.771)
Nighttime awakening	7.3	
Loss of interest	5.7	
Weight loss		
Weight loss	52.5	0.923 (0.896-0.953)
F7 gamma relative	5.1	
Fp2 delta relative	4.4	
Suicidal thoughts		
Suicidal thoughts	51.4	0.896 (0.873-0.923)
Agitation	5.5	
Appetite changes	4.5	
Nighttime awakening		
Nighttime awakening	49.0	0.786 (0.761-0.817)
Energy loss	5.5	
Diurnal variation	5.4	
Agitation		
Agitation	47.1	0.789 (0.759-0.822)
Unreality and nihilism	3.0	
F8 theta relative	2.9	
Appetite change		
Appetite changes	45.2	0.863 (0.84-0.886)
F3 alpha absolute	2.4	
Fp2 theta absolute	2.4	
Loss of interest		
Loss of interest	44.5	0.679 (0.647-0.710)
Energy loss	8.4	
Appetite changes	5.2	
Psychomotor retardation		
Psychomotor retardation	42.5	0.863 (0.833-0.893)
P4 alpha absolute	2.7	
Suicidal thoughts	2.1	
Unreality and nihilism		
Unreality and nihilism	40.9	0.951 (0.932-0.976)
T7-T3 beta relative ratio	4.7	
F7 beta relative	3.3	
Worrying		
Worrying	40.8	0.721 (0.688-0.751)
Psychomotor retardation	7.0	
F4 gamma absolute	6.6	
Libido loss		
Libido loss	40.8	0.777 (0.747-0.807)
T8-T4 theta and alpha relative ratio	3.3	
P8-T6 alpha relative ratio	3.1	
Obsessive thoughts		
Obsessive thoughts	39.8	0.882 (0.856-0.911)
Nighttime awakening	9.2	
O1 theta absolute	7.3	
Paranoia		
Paranoia	39.7	0.918 (0.888-0.951)
Oz alpha absolute	6.7	
T8-T4 beta absolute ratio	4.7	
Health preoccupation		
Health preoccupation	39.0	0.908 (0.872-0.944)
C4 theta relative	6.8	
T8-T4 beta relative ratio	4.9	
Diurnal variation		
Diurnal variation	38.3	0.831 (0.807-0.857)
Cp4 gamma absolute	4.4	
T7-T3 delta absolute ratio	4.1	
Energy loss		
Energy loss	32.5	0.676 (0.637-0.713)
Pz delta relative	4.1	
FCz delta relative	3.4	
Loss of insight		
Loss of insight	27.3	0.963 (0.939-1.000)
O1 delta absolute	18.8	
Oz delta absolute	6.7	
Depressed mood		
Depressed mood	23.2	0.662 (0.633-0.700)
P4 alpha absolute	3.9	
P4 theta-alpha absolute ratio	2.7	

Abbreviations: EEG, electroencephalograph; HRSD-21, 21-Item Hamilton Rating Scale for Depression.

^a^ The 3 most important features for each model, and their relative contributions computed using Shapley values, are reported.

### Feature Importance

The most important feature for each symptom was the score of that symptom at baseline. The importance of the baseline symptom score was higher than 20% on all symptoms, with the highest association for waking early (64.3%), and lowest association for depressed mood (23.2%) (Table 2). On 10 symptoms, prediction of improvement in a particular symptom involved associations from other symptoms as 1 of the 3 most important features, with the highest association of nighttime awakening (9.2% importance) with the prediction of improvement on the obsessive thoughts symptom.

The importance of any single EEG feature was higher than 5% for prediction of 7 symptoms (trouble sleeping, weight loss, agitation, worrying, obsessive thoughts, health preoccupation, and loss of insight), indicating the potential independent associations of pretreatment EEG. The most important EEG features were the absolute delta band power at the occipital electrode sites (O1, 18.8%; and Oz, 6.7%) for loss of insight (Table 2). Other notable EEG features included absolute occipital (O1) theta power for predicting improvement in obsessive thoughts (7.3%), relative central (C4) theta power for improvement in health preoccupation (6.8%), absolute temporal (T7 and T3) alpha power for improvement in trouble sleeping (6.7%), absolute occipital (Oz) alpha power for improvement in paranoia (6.7%), and absolute frontal (F4) gamma power for improvement in worrying (6.6%). The associations of the most important features for each symptom are detailed in Table 2.

### Using Both EEG and Baseline Symptoms vs Using Baseline Symptoms Alone

Over and above the use of baseline symptom scores alone, the use of both EEG and baseline symptom features produced a significant increase in the C index for improvement in 4 symptoms, including energy loss (C index increase, 0.035 [95% CI, 0.011-0.059]), appetite changes (C index increase, 0.017 [95% CI, 0.003-0.030]), psychomotor retardation (C index increase, 0.020 [95% CI, 0.008-0.032]), and loss of insight (C index increase, 0.012 [95% CI, 0.001-0.020]) (Table 3). On the *R*^2^ metric, for loss of insight, the use of both EEG and baseline symptom features produced an *R*^2^ of 0.551 (95% CI, 0.473-0.639), significantly higher than the *R*^2^ of 0.375 (95% CI, 0.31-0.448) produced by the use of the baseline symptom features alone. The differences for individual symptoms are reported in Table 3 and the absolute performances under both conditions are detailed in eTables 2, 3, 4, 5, 6, and 7 in the Supplement.

**Table 3.** Difference in C Index on the Prediction Task Using Combinations of HRSD-21 and EEG Features^a^

Item	Symptom	Difference between C index of baseline HRSD-21 features with EEG features and C index of HRSD-21 features without EEG features (95% CI)
1	Depressed mood	0.016 (−0.007 to 0.041)
2	Self-critical	0.000 (0.000 to 0.000)
3	Suicidal thoughts	0.021 (0.000 to 0.041)
4	Trouble sleeping	0.003 (−0.017 to 0.024)
5	Nighttime awakening	0.000 (0.000 to 0.000)
6	Waking early	0.000(0.000 to 0.000)
7	Loss of interest	0.000 (0.000 to 0.000)
8	Psychomotor retardation	0.020 (0.008 to 0.032)
9	Agitation	0.004 (−0.006 to 0.014)
10	Worrying	0.000 (−0.013 to 0.014)
11	Physical anxiety	0.001 (−0.010 to 0.013)
12	Appetite changes	0.017 (0.003 to 0.030)
13	Energy loss	0.035 (0.011 to 0.059)
14	Libido loss	0.011 (−0.004 to 0.024)
15	Health preoccupation	0.009 (−0.011 to 0.030)
16	Weight loss	0.006 (−0.011 to 0.021)
17	Loss of insight	0.012 (0.001 to 0.020)
18	Diurnal variation	0.013 (−0.001 to 0.026)
19	Unreality and nihilism	0.014 (−0.003 to 0.029)
20	Paranoia	0.018 (−0.005 to 0.041)
21	Obsessive thoughts	0.023 (−0.000 to 0.044)

Abbreviations: EEG, electroencephalograph; HRSD-21, 21-Item Hamilton Rating Scale for Depression.

^a^ Positive means that performance was higher with both sets of features included.

### Association of Treatment Group

There was no significant increase detected in the C index of any of the 21 items with the inclusion of the treatment group feature. The performances of the models for individual symptoms are reported in the eFigure and eTable 8 in the Supplement.

## Discussion

In this study, we developed a machine learning algorithm, ElecTreeScore, to evaluate the association of objective EEG measures acquired before treatment with the prediction of acute antidepressant response for individual symptoms of depression. Under this approach, we took into account the important associations between baseline symptom severity and treatment-associated change in symptoms and considered the association of EEG features in their own right and to what extent EEG features have a meaningful association with outcomes in addition to symptom severity.

Our machine learning approach resulted in 3 main findings. First, we found that different specific topologic characteristics and frequencies of neural activity assessed by the EEG were important for the prediction of antidepressant-associated improvement in specific symptoms in models with high discriminative performance. Second, although we found that baseline scores for individual symptoms of depression are strong predictors by themselves, as expected, we also found that EEG features add 5% or more in importance to the discriminative performance for 7 of the symptoms: trouble sleeping, weight loss, agitation, worrying, obsessive thoughts, health preoccupation, and loss of insight. Third, we demonstrated the value of the pretreatment EEG features in predicting improvement in a subset of specific depressive symptoms—loss of insight, energy loss, appetite changes, and psychomotor retardation—significantly better than with pretreatment symptom severity alone.

As expected, the most important feature was the score of the symptom at baseline, as seen when comparing the discriminative performance of training on only the EEG features and adding in the HRSD survey scores as inputs. However, our machine learning model suggests that EEG features are meaningfully associated with predicting individual symptom improvement both in combination with baseline symptom severity and over and above symptom severity as independent predictors. To identify independent predictors, we evaluated the addition of EEG features to baseline symptom severity and, in this model, 4 categories saw a significant increase in discriminative power: energy loss, psychomotor retardation, appetite changes, and loss of insight. Previous studies, with few exceptions,^35^ have focused on using EEG features to predict response or remission, which are defined by differences in summed symptom scores,^24,36,37^ and have yielded mixed outcomes.^20,22^ Electroencephalographic features that predict the change in summed symptom scores may not be replicated across populations of depression in which the primary depressive symptoms are highly heterogenous; thus, our findings offer an indication that the use of individual symptoms may be one means to address the replication gap in evaluating the potential value of EEG biomarkers of treatment outcomes in future studies. This approach might also help determine if EEG features add value to the previous suggestion that symptoms may have a differential rate of improvement.^10,13^

Our results expand our growing knowledge of the neurobiology of depression by revealing the relative importance of specific EEG markers in predicting treatment-associated changes in specific symptom domains beyond the association of baseline symptoms alone. In particular, we observed that prediction of treatment-associated changes in psychomotor retardation, energy loss, appetite changes, and loss of insight are improved significantly with the inclusion of EEG features, with parietal alpha power providing the largest association for psychomotor retardation, parietal delta power providing the largest association for energy loss, frontal alpha power providing the largest association for appetite changes, and occipital delta power providing the largest association for loss of insight. These associations of baseline EEG markers build on findings for the implication of EEG marker abnormalities in depression and point to future lines of investigation for treatment trials. For example, hedonic hunger signals and altered eating behaviors have been previously associated with frontal alpha power^38^; our finding that occipital delta power is substantially associated with improvement in the symptom of loss of insight is in accordance with prior work showing altered delta power in depression.^39,40^ Loss of insight is implicated in higher risks of suicide and self-harm^41^ and delayed treatment seeking^42,43,44,45^; in this context, we speculate that knowing about pretreatment delta power might be of use in identifying an important feature for treatment in patients at risk of a poor prognosis. Energy loss and psychomotor retardation are also implicated in anhedonic forms of depression that have a poor prognosis. Together, these findings suggest that changes in specific pretreatment EEG features are not just implicated in the pathophysiological characteristics of depression, but may be associated with antidepressant response in specific symptoms. Our models therefore generate testable hypotheses about the potential mechanisms of symptom change over time that may be tested in future studies.

In our exploratory analyses, we did not find evidence that the inclusion of treatment group significantly improved model performance. This finding suggests that the EEG markers associated with changes in symptom scores were general predictors of treatment outcome rather than differentiating response among the treatment types. In a previous functional neuroimaging study of a subset of this sample, resting-state predictors were also robust, general predictors of treatment outcome.^46^ By contrast, specific task-evoked markers have been found to be differential predictors of response to different treatments.^47,48^ Therefore, future studies may investigate task-evoked EEG markers in determining differential treatment response.

Although EEG offers one of the most proximal measures of neural function, there have been barriers to its use as a pertinent objective predictor of antidepressant response. Foundational studies using EEG markers for the prediction of depression treatment response have necessarily relied on small samples, with insufficient power for estimating the robustness of predictive models.^26,35,36,37^ A recent meta-analysis reported that only 6 of 71 studies of EEG markers and antidepressant outcomes were studied with cross-validation or another out-of-sample verification.^17^ As the field develops, and the opportunity for acquiring larger samples becomes feasible, we can further address the understandable power constraints of these foundational studies. Prior treatment studies have also understandably focused on response outcomes based on averaged symptom ratings. It is notable that prediction by EEG markers in our model was specific to individual symptoms. Evaluation of individual symptoms (rather than summed severity scores) may thus be valuable in the future application of machine learning with biomarkers such as those derived from EEG recordings. Because direct symptom measurement is increasingly included as a routine part of clinical psychiatry,^49^ it is feasible to consider how clinicians of the future will have access to symptom profiles linked to biomarkers through machine learning algorithms. A first-use case might be for detection of high-risk patients; for example, those with symptoms such as loss of touch with reality (loss of insight, and unreality and nihilism) are included in primary care guidelines as an indication of elevated suicide risk^41^ and for which same-day mental health care is recommended.

Regarding clinical applications in treatment management, our models provide a first proof of principle that noninvasive neurobiological markers and pretreatment symptom assessments may be used to determine whether specific symptom domains are likely to persist with standard antidepressant treatment. Currently, only approximately 30% of patients recover with the first antidepressant treatment attempted, and approximately only one-half of patients show some symptom response.^29^ Physicians lack algorithmic support for determining who will respond to available treatments, as well as a means to select between them. To reduce patients’ burden of trying multiple rounds of unsuccessful treatments (often associated with worsening of symptom severity), models such as ours, when validated in prospective clinical settings, could be used to predict outcomes ahead of time. Future studies may attempt to recruit individuals with a more constrained definition of baseline severity in specific symptom domains (eg, balanced samples with exceptionally high or low scores on 1 symptom domain) to determine more directly the maximum additional benefit of EEG markers once the variance of baseline severity has been more constrained. These results bring us closer to a future of using predictive models to guide individualized treatment strategies on the basis of specific symptom domains in combination with objective markers.

### Limitations

This study has several important limitations. First, we only explored the interactions of markers from EEGs recorded with eyes closed; this decision was based on previous literature, but using EEGs recorded with eyes open is an area of further investigation. Second, while we found that the model was a general predictor of response across treatments, we did not perform a subgroup analysis of performance on each treatment or analyze the performance of models separately trained on each treatment, which may be able to capture adverse effects associated with certain antidepressants. Third, we did not evaluate the performance of our algorithm for other treatments for depression (such as repetitive transcranial magnetic stimulation, for which EEG markers may also be able to predict response)^17^ or for treatments that add a second medication to an initial, ineffective antidepressant drug.^42^ Fourth, the absence of a placebo means that we are unable to determine with our present models whether the changes in symptoms observed are specifically caused by the antidepressant treatments used, but future studies may use our modeling approach to address this possibility in placebo-controlled trials. Fifth, our models have been validated retrospectively, and on the same data set (iSPOT-D) that the model has been developed, necessarily given the limited availability of large data sets with pretreatment EEG recordings with associated pretreatment and posttreatment scores. Future studies should investigate the utility of ElecTreeScore in prospective data sets to advance the translational goal of application for clinical use.

## Conclusions

A machine learning model was developed to predict improvement of specific symptoms associated with antidepressants using symptom ratings and EEG measures acquired at the pretreatment baseline. We found that the model had high discriminative performance for identifying improvement in specific symptoms, reflected in high C index scores of 0.8 or higher on 12 of 21 clinician-rated symptoms. The most important feature in the prediction of symptom improvements was the symptom score at baseline, whereas EEG features had smaller but meaningful associations with the prediction of specific symptom improvements. Overall, our findings build on prior work in 2 key ways: first, by demonstrating that predictive models can capitalize on established roles for using EEG markers to quantify neural activity in psychiatric illness to predict treatment-associated changes over time, and second, by explicitly using individual symptoms as independent outcome variables, to parse the extreme heterogeneity of major depression. Future work should investigate the performance of this model prospectively and in application of independent samples and clinical settings.

## Notes

Supplement. **eTable 1.** Mean and Standard Deviation Scores for Each of the 21 Items on the HRSD21 Report at the Baseline Visit, the Week 8 Clinical Visit on the Entire Dataset **eTable 2.** The C-Indices of the Machine Learning Models on the Improvement Prediction (Reduction in HRSD Score) Using Baseline HRSD Features With and Without the EEG Features (Positive Means That Performance Was Higher With the EEG Features Included) **eTable 3.** The C-Indices of the Machine Learning Models on the Improvement Prediction Task (Reduction in HRSD Score) Using Baseline EEG Features With and Without HRSD Features (Positive Means That Performance Was Higher With the HRSD Features Included) **eTable 4.** Comparison of R2 Score Computed on Calibrated Machine Learning Model Predictions **eTable 5.** Comparison of MAE Score Computed on Calibrated Machine Learning Model Predictions **eTable 6.** Comparison of Regression Slope Computed on Calibrated Machine Learning Model Predictions **eTable 7.** Comparison of Regression Intercept Computed on Calibrated Machine Learning Model Predictions **eTable 8.** Short Notation of HRSD Targets, Used in eFigure **eFigure.** Visualization of Comparison of Confidence Interval Between Models That Use One-Hot Encoded Treatment Arm as Input and Models That Do Not Use Treatment Information **eAppendix.** Supplementary Information
