# Error in Methods Section

**DOI:** 10.1001/jamanetworkopen.2020.16001

**Published:** 2020-07-20

In the Original Investigation titled “Evaluation of a Machine Learning Model Based on Pretreatment Symptoms and Electroencephalographic Features to Predict Outcomes of Antidepressant Treatment in Adults With Depression: A Prespecified Secondary Analysis of a Randomized Clinical Trial,”^1^ published June 22, 2020, the third-to-last sentence of the first paragraph of the Statistical Analysis subection of the Methods section should read: “A linear regression with an L1 regularization coefficient of 0.01 was chosen to be the calibration model.” This article has been corrected.
