# Supplementary material for: Evaluation of a Machine Learning Model Based on Pretreatment Symptoms and Electroencephalographic Features to Predict Outcomes of Antidepressant Treatment in Adults With Depression: A Prespecified Secondary Analysis of a Randomized Clinical Trial
Source: JAMA Netw Open. 2020 Jun 22;3(6):e206653. doi: 10.1001/jamanetworkopen.2020.6653 (PMC7309440; doi:10.1001/jamanetworkopen.2020.6653)
Supplement: Supplement. — eTable 1. Mean and Standard Deviation Scores for Each of the 21 Items on the HRSD21 Report at the Baseline Visit, the Week 8 Clinical Visit on the Entire Dataset eTable 2. The C-Indices of the Machine Learning Models on the Improvement Prediction (Reduction in HRSD Score) Using Baseline HRSD Features With and Without the EEG Features (Positive Means That Performance Was Higher With the EEG Features Included) eTable 3. The C-Indices of the Machine Learning Models on the Improvement Prediction Task (Reduction in HRSD Score) Using Baseline EEG Features With and Without HRSD Features (Positive Means That Performance Was Higher With the HRSD Features Included) eTable 4. Comparison of R2 Score Computed on Calibrated Machine Learning Model Predictions eTable 5. Comparison of MAE Score Computed on Calibrated Machine Learning Model Predictions eTable 6. Comparison of Regression Slope Computed on Calibrated Machine Learning Model Predictions eTable 7. Comparison of Regression Intercept Computed on Calibrated Machine Learning Model Predictions eTable 8. Short Notation of HRSD Targets, Used in eFigure eFigure. Visualization of Comparison of Confidence Interval Between Models That Use One-Hot Encoded Treatment Arm as Input and Models That Do Not Use Treatment Information eAppendix. Supplementary Information [file jamanetwopen-3-e206653-s001.pdf]

Supplementary Online Content

Rajpurkar P, Yang J, Dass N, et al. Evaluation of a machine learning model based on pretreatment symptoms and electroencephalographic features to predict outcomes of antidepressant treatment in adults with depression: a prespecified secondary analysis of a randomized clinical trial. *JAMA Netw Open.* 2020;3(6):e206653. doi:10.1001/jamanetworkopen.2020.6653

eTable 1. Mean and Standard Deviation Scores for Each of the 21 Items on the HRSD21 Report at the Baseline Visit, the Week 8 Clinical Visit on the Entire Dataset
eTable 2. The C-Indices of the Machine Learning Models on the Improvement Prediction (Reduction in HRSD Score) Using Baseline HRSD Features With and Without the EEG Features (Positive Means That Performance Was Higher With the EEG Features Included)
eTable 3. The C-Indices of the Machine Learning Models on the Improvement Prediction Task (Reduction in HRSD Score) Using Baseline EEG Features With and Without HRSD Features (Positive Means That Performance Was Higher With the HRSD Features Included)
eTable 4. Comparison of R2 Score Computed on Calibrated Machine Learning Model Predictions
eTable 5. Comparison of MAE Score Computed on Calibrated Machine Learning Model Predictions
eTable 6. Comparison of Regression Slope Computed on Calibrated Machine Learning Model Predictions
eTable 7. Comparison of Regression Intercept Computed on Calibrated Machine Learning Model Predictions
eTable 8. Short Notation of HRSD Targets, Used in eFigure
eFigure. Visualization of Comparison of Confidence Interval Between Models That Use One-Hot Encoded Treatment Arm as Input and Models That Do Not Use Treatment Information
eAppendix. Supplementary Information

This supplementary material has been provided by the authors to give readers additional information about their work.

© 2020 Rajpurkar P et al. *JAMA Network Open.*

eTable 1. Mean and Standard Deviation Scores for Each of the 21 Items on the HRSD21 Report at the Baseline Visit, the Week 8 Clinical Visit on the Entire Dataset

Item	Symptom	Baseline score mean (std)	Post-treatment score mean (std)
1	Depressed Mood	2.74 (0.62)	1.19 (0.87)
2	Self-Critical	1.95 (0.87)	0.81 (0.86)
3	Suicidal Thoughts	0.60 (0.73)	0.17 (0.47)
4	Trouble Sleeping	1.30 (0.85)	0.65 (0.77)
5	Nighttime Awakening	1.25 (0.80)	0.62 (0.74)
6	Waking Early	1.06 (0.83)	0.48 (0.69)
7	Loss of Interest	2.65 (0.74)	1.08 (0.97)
8	Psychomotor Retardation	0.95 (0.83)	0.33 (0.59)
9	Agitation	1.31 (1.01)	0.60 (0.80)
10	Worrying	2.09 (0.84)	0.94 (0.86)
11	Physical Anxiety	1.33 (0.96)	0.58 (0.73)
12	Appetite Changes	0.72 (0.73)	0.29 (0.54)
13	Energy Loss	1.64 (0.56)	0.74 (0.70)
14	Libido Loss	1.11 (0.82)	0.64 (0.78)
15	Health Preoccupation	0.33 (0.62)	0.13 (0.38)
16	Weight Loss	0.43 (0.69)	0.14 (0.41)
17	Loss of Insight	0.11 (0.32)	0.04 (0.19)
18	Diurnal Variation	0.88 (0.83)	0.49 (0.7)
19	Unreality and Nihilism	0.36 (0.74)	0.11 (0.37)
20	Paranoia	0.29 (0.52)	0.11 (0.33)
21	Obsessive Thoughts	0.27 (0.46)	0.19 (0.42)

© 2020 Rajpurkar P et al. *JAMA Network Open.*

eTable 2. The C-Indices of the Machine Learning Models on the Improvement Prediction (Reduction in HRSD Score) Using Baseline HRSD Features With and Without the EEG Features (Positive Means That Performance Was Higher With the EEG Features Included)

Item	Symptom	C-index of baseline HRSD Symptoms without EEG (95% CI)	C-index of baseline HRSD Symptoms with EEG (95% CI)
1	Depressed Mood	0.647 (0.619, 0.677)	0.663 (0.634, 0.692)
2	Self-Critical	0.743 (0.72, 0.768)	0.743 (0.72, 0.768)
3	Suicidal Thoughts	0.875 (0.852, 0.9)	0.896 (0.872, 0.92)
4	Trouble Sleeping	0.769 (0.744, 0.793)	0.772 (0.75, 0.796)
5	Nighttime Awakening	0.786 (0.762, 0.81)	0.786 (0.764, 0.811)
6	Waking Early	0.833 (0.813, 0.854)	0.833 (0.813, 0.854)
7	Loss of Interest	0.677 (0.649, 0.703)	0.677 (0.65, 0.707)
8	Psychomotor Retardation	0.844 (0.82, 0.867)	0.864 (0.843, 0.887)
9	Agitation	0.783 (0.759, 0.807)	0.787 (0.763, 0.811)
10	Worrying	0.718 (0.693, 0.744)	0.718 (0.691, 0.745)
11	Physical Anxiety	0.802 (0.78, 0.823)	0.804 (0.782, 0.826)
12	Appetite Changes	0.844 (0.822, 0.866)	0.861 (0.839, 0.882)
13	Energy Loss	0.643 (0.613, 0.674)	0.677 (0.648, 0.706)
14	Libido Loss	0.765 (0.741, 0.789)	0.776 (0.753, 0.798)
15	Health Preoccupation	0.9 (0.869, 0.932)	0.909 (0.878, 0.941)
16	Weight Loss	0.917 (0.896, 0.939)	0.922 (0.902, 0.943)
17	Loss of Insight	0.951 (0.92, 0.987)	0.962 (0.937, 0.995)
18	Diurnal Variation	0.819 (0.798, 0.84)	0.831 (0.811, 0.854)
19	Unreality and Nihilism	0.936 (0.914, 0.964)	0.951 (0.934, 0.972)
20	Paranoia	0.899 (0.872, 0.93)	0.917 (0.893, 0.945)
21	Obsessive Thoughts	0.859 (0.83, 0.889)	0.882 (0.859, 0.908)

© 2020 Rajpurkar P et al. *JAMA Network Open.*

eTable 3. The C-Indices of the Machine Learning Models on the Improvement Prediction Task (Reduction in HRSD Score) Using Baseline EEG Features With and Without HRSD Features (Positive Means That Performance Was Higher With the HRSD Features Included)

Item	Symptom	C-index of baseline EEG (95% CI)	C-index of baseline HRSD Symptoms with EEG (95% CI)
1	Mood Depressed	0.558 (0.526, 0.587)	0.663 (0.634, 0.692)
2	Self-Critical	0.534 (0.501, 0.566)	0.743 (0.72, 0.768)
3	Suicidal Thoughts	0.547 (0.509, 0.581)	0.896 (0.872, 0.92)
4	Trouble Sleeping	0.54 (0.509, 0.573)	0.772 (0.75, 0.796)
5	Nighttime Awakening	0.522 (0.49, 0.555)	0.786 (0.764, 0.811)
6	Waking Early	0.518 (0.482, 0.551)	0.833 (0.813, 0.854)
7	Loss of Interest	0.528 (0.498, 0.56)	0.677 (0.65, 0.707)
8	Psychomotor Retardation	0.528 (0.491, 0.563)	0.864 (0.843, 0.887)
9	Agitation	0.562 (0.529, 0.597)	0.787 (0.763, 0.811)
10	Worrying	0.562 (0.53, 0.591)	0.718 (0.691, 0.745)
11	Physical Anxiety	0.523 (0.493, 0.553)	0.804 (0.782, 0.826)
12	Appetite Changes	0.553 (0.517, 0.589)	0.861 (0.839, 0.882)
13	Energy Loss	0.546 (0.509, 0.581)	0.677 (0.648, 0.706)
14	Libido Loss	0.531 (0.494, 0.569)	0.776 (0.753, 0.798)
15	Health Preoccupation	0.506 (0.46, 0.554)	0.909 (0.878, 0.941)
16	Weight Loss	0.549 (0.508, 0.589)	0.922 (0.902, 0.943)
17	Loss of Insight	0.54 (0.469, 0.609)	0.962 (0.937, 0.995)
18	Diurnal Variation	0.536 (0.498, 0.572)	0.831 (0.811, 0.854)
19	Unreality and Nihilism	0.593 (0.549, 0.637)	0.951 (0.934, 0.972)
20	Paranoia	0.543 (0.498, 0.59)	0.917 (0.893, 0.945)
21	Obsessive Thoughts	0.628 (0.575, 0.682)	0.882 (0.859, 0.908)

© 2020 Rajpurkar P et al. *JAMA Network Open.*

eTable 4. Comparison of R2 Score Computed on Calibrated Machine Learning Model Predictions. Values in bracket represent lower and upper bound of 95% confidence interval.

Item	Symptom	Symptom+EEG R2	Symptom R2	EEG R2
1	Depressed mood	0.09 (0.005, 0.185)	0.119 (0.063, 0.169)	-0.084 (-0.143, -0.012)
2	Self critical	0.275 (0.19, 0.366)	0.306 (0.24, 0.37)	-0.031 (-0.094, 0.034)
3	Suicidal thoughts	0.483 (0.395, 0.575)	0.499 (0.428, 0.581)	-0.001 (-0.013, 0.01)
4	Trouble sleeping	0.324 (0.275, 0.385)	0.355 (0.317, 0.399)	0.01 (-0.009, 0.028)
5	Nighttime awakening	0.366 (0.307, 0.416)	0.358 (0.291, 0.434)	-0.02 (-0.056, 0.023)
6	Waking early	0.444 (0.391, 0.501)	0.444 (0.383, 0.497)	-0.055 (-0.105, -0.01)
7	Loss of interest	0.136 (0.059, 0.218)	0.12 (0.024, 0.197)	-0.014 (-0.037, 0.008)
8	Psychomotor retardation	0.462 (0.387, 0.545)	0.462 (0.399, 0.552)	-0.01 (-0.028, 0.007)
9	Agitation	0.343 (0.272, 0.43)	0.301 (0.224, 0.398)	-0.068 (-0.13, 0.021)
10	Worrying	0.259 (0.192, 0.341)	0.255 (0.181, 0.315)	0.009 (-0.022, 0.038)
11	Physical anxiety	0.405 (0.344, 0.478)	0.361 (0.283, 0.462)	-0.037 (-0.076, 0.023)
12	Appetite changes	0.501 (0.44, 0.576)	0.481 (0.406, 0.55)	-0.025 (-0.061, 0.014)
13	Energy loss	0.132 (0.081, 0.185)	0.106 (0.056, 0.181)	-0.014 (-0.051, 0.023)
14	Libido loss	0.325 (0.279, 0.377)	0.326 (0.287, 0.369)	-0.052 (-0.106, 0.002)
15	Health preoccupation	0.6 (0.514, 0.708)	0.605 (0.531, 0.699)	-0.024 (-0.065, 0.024)
16	Weight loss	0.645 (0.586, 0.697)	0.63 (0.573, 0.711)	-0.01 (-0.024, 0.006)
17	Loss of insight	0.551 (0.473, 0.639)	0.375 (0.31, 0.448)	-0.002 (-0.004, 0.008)
18	Diurnal variation	0.398 (0.347, 0.45)	0.374 (0.318, 0.437)	-0.054 (-0.109, -0.003)
19	Unreality and nihilism	0.732 (0.659, 0.812)	0.606 (0.54, 0.681)	-0.019 (-0.06, 0.029)
20	Paranoia	0.525 (0.453, 0.626)	0.466 (0.389, 0.557)	-0.002 (-0.005, 0.004)
21	Obsessive thoughts	0.259 (0.198, 0.333)	0.272 (0.206, 0.36)	0.002 (-0.009, 0.021)

© 2020 Rajpurkar P et al. *JAMA Network Open.*

eTable 5. Comparison of MAE Score Computed on Calibrated Machine Learning Model Predictions. Values in bracket represent lower and upper bound of 95% confidence interval.

Item	Symptom	Symptom+EEG MAE	Symptom MAE	EEG MAE
1	Depressed mood	0.728 (0.676, 0.773)	0.747 (0.697, 0.78)	0.816 (0.775, 0.87)
2	Self critical	0.733 (0.689, 0.778)	0.716 (0.678, 0.75)	0.859 (0.814, 0.9)
3	Suicidal thoughts	0.354 (0.324, 0.388)	0.375 (0.339, 0.408)	0.6 (0.568, 0.631)
4	Trouble sleeping	0.558 (0.524, 0.595)	0.54 (0.499, 0.579)	0.736 (0.7, 0.773)
5	Nighttime awakening	0.577 (0.54, 0.615)	0.594 (0.551, 0.629)	0.763 (0.716, 0.806)
6	Waking early	0.544 (0.503, 0.581)	0.555 (0.514, 0.586)	0.786 (0.733, 0.823)
7	Loss of interest	0.856 (0.797, 0.906)	0.872 (0.818, 0.925)	0.934 (0.885, 0.987)
8	Psychomotor Retardation	0.416 (0.382, 0.446)	0.408 (0.377, 0.437)	0.66 (0.625, 0.697)
9	Agitation	0.555 (0.517, 0.594)	0.604 (0.563, 0.65)	0.755 (0.708, 0.795)
10	Worrying	0.693 (0.658, 0.738)	0.715 (0.684, 0.752)	0.801 (0.756, 0.86)
11	Physical Anxiety	0.561 (0.527, 0.609)	0.569 (0.517, 0.608)	0.769 (0.724, 0.813)
12	Appetite changes	0.412 (0.371, 0.454)	0.435 (0.399, 0.461)	0.676 (0.635, 0.71)
13	Energy loss	0.562 (0.526, 0.604)	0.574 (0.535, 0.607)	0.572 (0.537, 0.611)
14	Libido loss	0.556 (0.525, 0.599)	0.557 (0.518, 0.595)	0.714 (0.666, 0.766)
15	Health preoccupation	0.197 (0.174, 0.217)	0.198 (0.169, 0.223)	0.391 (0.356, 0.428)
16	Weight loss	0.284 (0.259, 0.308)	0.264 (0.231, 0.289)	0.554 (0.515, 0.597)
17	Loss of insight	0.127 (0.108, 0.139)	0.15 (0.136, 0.166)	0.167 (0.145, 0.189)
18	Diurnal variation	0.488 (0.454, 0.52)	0.49 (0.461, 0.525)	0.661 (0.623, 0.709)
19	Unreality and nihilism	0.296 (0.272, 0.33)	0.313 (0.281, 0.336)	0.48 (0.431, 0.523)
20	Paranoia	0.233 (0.212, 0.261)	0.257 (0.235, 0.28)	0.348 (0.322, 0.377)
21	Obsessive thoughts	0.214 (0.187, 0.239)	0.224 (0.2, 0.246)	0.239 (0.205, 0.267)

© 2020 Rajpurkar P et al. *JAMA Network Open.*

eTable 6. Comparison of Regression Slope Computed on Calibrated Machine Learning Model Predictions. Values in bracket represent lower and upper bound of 95% confidence interval.

Item	Symptom	Symptom+EEG Regression Slope	Symptom Regression Slope	EEG Regression Slope
1	Depressed mood	0.176 (0.131, 0.219)	0.109 (0.078, 0.13)	0.011 (-0.014, 0.035)
2	Self critical	0.332 (0.298, 0.363)	0.339 (0.304, 0.378)	0.037 (0.014, 0.06)
3	Suicidal thoughts	0.531 (0.488, 0.585)	0.47 (0.426, 0.528)	0.003 (-0.002, 0.009)
4	Trouble sleeping	0.344 (0.308, 0.382)	0.349 (0.32, 0.375)	0.016 (0.005, 0.025)
5	Nighttime awakening	0.369 (0.334, 0.401)	0.384 (0.347, 0.419)	0.016 (-0.001, 0.033)
6	Waking early	0.443 (0.415, 0.478)	0.452 (0.417, 0.484)	0.007 (-0.011, 0.025)
7	Loss of interest	0.214 (0.166, 0.259)	0.197 (0.156, 0.232)	0.004 (-0.007, 0.012)
8	Psychomotor retardation	0.462 (0.428, 0.499)	0.469 (0.423, 0.508)	0.002 (-0.006, 0.009)
9	Agitation	0.393 (0.353, 0.434)	0.319 (0.283, 0.356)	0.027 (0.004, 0.055)
10	Worrying	0.295 (0.254, 0.335)	0.267 (0.224, 0.299)	0.02 (0.007, 0.038)
11	Physical anxiety	0.407 (0.372, 0.454)	0.407 (0.36, 0.459)	0.033 (0.005, 0.057)
12	Appetite changes	0.5 (0.463, 0.536)	0.493 (0.453, 0.531)	0.008 (-0.012, 0.027)
13	Energy loss	0.142 (0.116, 0.165)	0.152 (0.122, 0.182)	0.019 (0.001, 0.035)
14	Libido loss	0.315 (0.282, 0.338)	0.306 (0.278, 0.335)	0.022 (-0.0, 0.045)
15	Health preoccupation	0.552 (0.493, 0.614)	0.575 (0.511, 0.638)	0.02 (0.001, 0.039)
16	Weight loss	0.627 (0.58, 0.677)	0.609 (0.556, 0.654)	-0.002 (-0.007, 0.005)
17	Loss of insight	0.439 (0.381, 0.488)	0.262 (0.227, 0.297)	-0.0 (-0.0, 0.0)
18	Diurnal variation	0.403 (0.369, 0.433)	0.373 (0.345, 0.405)	0.022 (-0.006, 0.05)
19	Unreality and nihilism	0.648 (0.597, 0.7)	0.636 (0.573, 0.687)	0.029 (0.003, 0.051)
20	Paranoia	0.49 (0.447, 0.536)	0.415 (0.379, 0.455)	-0.0 (-0.001, 0.0)
21	Obsessive thoughts	0.22 (0.176, 0.255)	0.255 (0.218, 0.298)	0.004 (-0.001, 0.009)

© 2020 Rajpurkar P et al. *JAMA Network Open.*

eTable 7. Comparison of Regression Intercept Computed on Calibrated Machine Learning Model Predictions. Values in bracket represent lower and upper bound of 95% confidence interval.

Item	Symptom	Symptom+EEG Regression Intercept	Symptom Regression Intercept	EEG Regression Intercept
1	Depressed mood	-1.26 (-1.348, -1.198)	-1.384 (-1.428, -1.342)	-1.519 (-1.558, -1.469)
2	Self critical	-0.743 (-0.813, -0.673)	-0.745 (-0.825, -0.676)	-1.077 (-1.115, -1.031)
3	Suicidal thoughts	-0.205 (-0.257, -0.159)	-0.241 (-0.284, -0.206)	-0.441 (-0.446, -0.436)
4	Trouble sleeping	-0.438 (-0.499, -0.382)	-0.44 (-0.492, -0.373)	-0.663 (-0.673, -0.652)
5	Nighttime awakening	-0.397 (-0.447, -0.355)	-0.374 (-0.422, -0.316)	-0.616 (-0.632, -0.594)
6	Waking early	-0.324 (-0.374, -0.265)	-0.303 (-0.351, -0.263)	-0.59 (-0.615, -0.566)
7	Loss of interest	-1.256 (-1.335, -1.182)	-1.272 (-1.387, -1.196)	-1.562 (-1.582, -1.541)
8	Psychomotor Retardation	-0.324 (-0.38, -0.278)	-0.33 (-0.381, -0.279)	-0.614 (-0.621, -0.606)
9	Agitation	-0.413 (-0.464, -0.361)	-0.513 (-0.577, -0.458)	-0.676 (-0.709, -0.65)
10	Worrying	-0.796 (-0.881, -0.74)	-0.835 (-0.899, -0.761)	-1.126 (-1.146, -1.106)
11	Physical anxiety	-0.425 (-0.478, -0.376)	-0.434 (-0.49, -0.364)	-0.704 (-0.74, -0.664)
12	Appetite changes	-0.225 (-0.278, -0.18)	-0.224 (-0.276, -0.178)	-0.46 (-0.475, -0.446)
13	Energy loss	-0.761 (-0.802, -0.726)	-0.741 (-0.793, -0.704)	-0.868 (-0.897, -0.845)
14	Libido loss	-0.334 (-0.377, -0.294)	-0.34 (-0.379, -0.303)	-0.478 (-0.499, -0.456)
15	Health preoccupation	-0.081 (-0.103, -0.059)	-0.082 (-0.111, -0.052)	-0.194 (-0.206, -0.183)
16	Weight loss	-0.107 (-0.153, -0.073)	-0.13 (-0.166, -0.087)	-0.309 (-0.313, -0.304)
17	Loss of insight	-0.051 (-0.065, -0.039)	-0.059 (-0.067, -0.05)	-0.079 (-0.079, -0.079)
18	Diurnal variation	-0.236 (-0.278, -0.192)	-0.233 (-0.28, -0.195)	-0.362 (-0.388, -0.335)
19	Unreality and nihilism	-0.096 (-0.13, -0.06)	-0.065 (-0.097, -0.024)	-0.253 (-0.269, -0.237)
20	Paranoia	-0.086 (-0.108, -0.062)	-0.094 (-0.119, -0.069)	-0.162 (-0.163, -0.162)
21	Obsessive thoughts	-0.077 (-0.092, -0.063)	-0.068 (-0.085, -0.053)	-0.086 (-0.089, -0.084)

© 2020 Rajpurkar P et al. *JAMA Network Open.*

eTable 8. Short Notation of HRSD Targets, Used in eFigure

Item	Symptom	Symptom Short Form
1	Depressed Mood	MOOD
2	Self-Critical	CRITICAL
3	Suicidal Thoughts	SUOUGHTS
4	Trouble Sleeping	TREEPING
5	Nighttime Awakening	WAKING
6	Waking Early	AWNEARLY
7	Loss of Interest	SPNGTIME
8	Psychomotor Retardation	REDATION
9	Agitation	AGTATION
10	Worrying	WORRYING
11	Physical Anxiety	PHMPTOMS
12	Appetite Changes	APPETITE
13	Energy Loss	ENERGY
14	Libido Loss	SETEREST
15	Health Preoccupation	HEOUGHTS
16	Weight Loss	WEHTLOSS
17	Loss of Insight	INSIGHT
18	Diurnal Variation	DIIATION
19	Unreality and Nihilism	DESATION
20	Paranoia	PARANOID
21	Obsessive Thoughts	OBSIONAL

© 2020 Rajpurkar P et al. *JAMA Network Open.*

eFigure. Visualization of Comparison of Confidence Interval Between Models That Use One-Hot Encoded Treatment Arm as Input and Models That Do Not Use Treatment Information

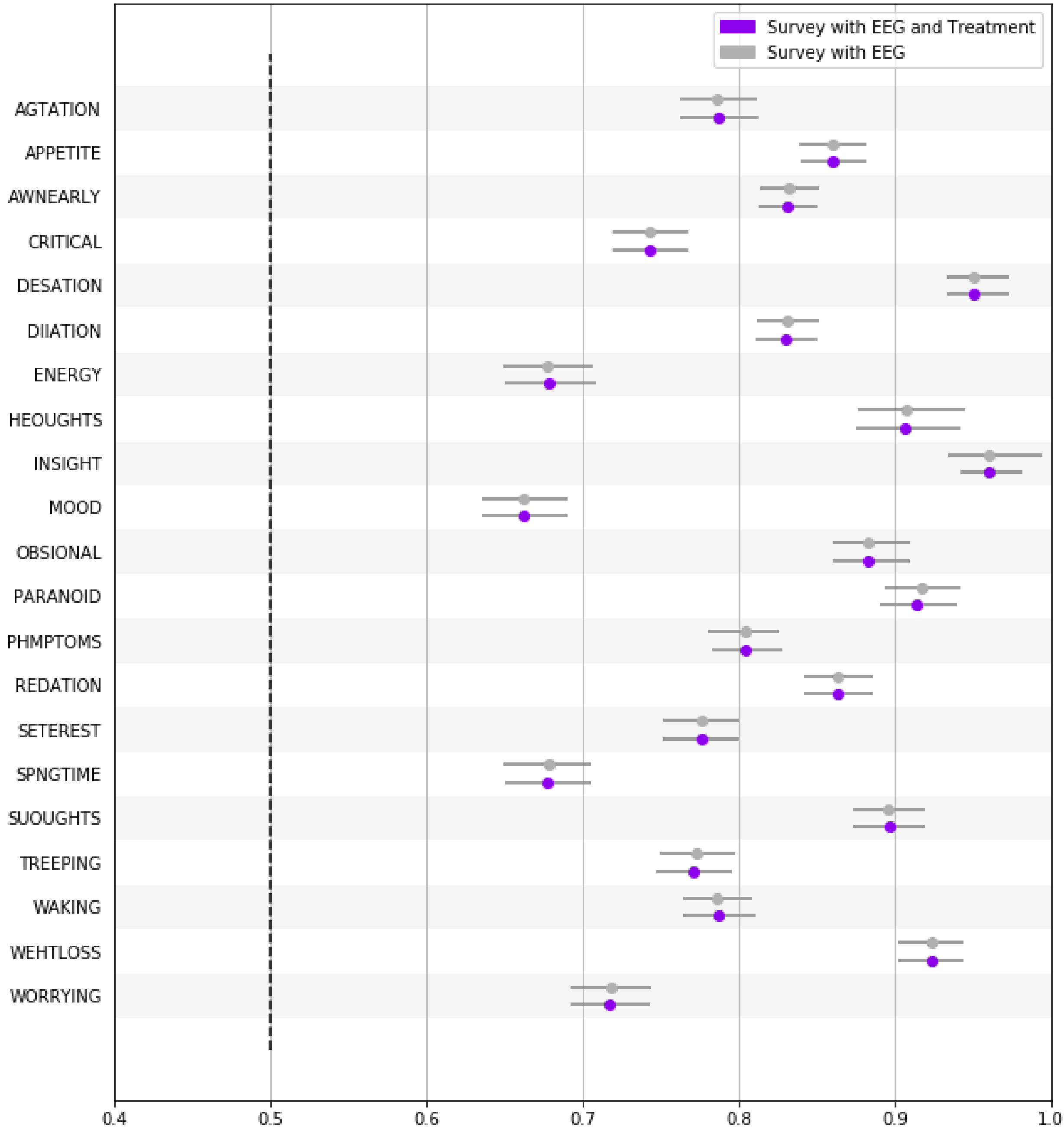

© 2020 Rajpurkar P et al. *JAMA Network Open.*

eAppendix. Supplementary Information

Stratified K-Fold Implementation

Since we are predicting differences and ratio of improvement in HRSD categories, there are often target classes that occur fewer than k times. This can be problematic because not all of the splits will contain the target classes in the train and test splits. This problem is exacerbated when the target value is continuous, leaving the definition of a target class ambiguous. To address this, we implement a modified stratified k-fold cross validation that merges target classes with neighboring classes to increase the number of folds where each target class is present in the train and test split. Our algorithm is as follows: find the target class that has the fewest number of occurrences, find the nearest neighbors on the left and right that have not been merged already, and assign the target class to the neighbor that has the smaller number of occurrences. In short, the selected neighbour now represents both the original class and the target class with insufficient number of occurrences. This process is repeated until there is no target class that has fewer than k occurences. This algorithm is only used to generate better splits, but the actual class values, for both the target class and the selected neighbour class, are not altered. Initial stratification for our modified k-fold is the same as a standard stratified k-fold algorithm, not correlated to categories outlined in Figure 2.

EEG Input Feature Search

To find the best EEG input features for each HRSD category, we perform a search over various combinations of input features by altering the bands, time windows, and relative or absolute power of the EEG features. Each feature is calculated at each of the 26 electrodes . We consider combinations of all 5 EEG bands: delta, theta, alpha, beta, and gamma. If only the alpha band is being used, the time window over which the bandpower is calculated is chosen from a subset of 24 seconds, 30 seconds, 40 seconds, 60 seconds, and the full 120 second

© 2020 Rajpurkar P et al. *JAMA Network Open.*

EEG sequence. If bands besides only the alpha band are being used, the time window over which the bandpower is calculated is fixed to the full 120 second EEG sequence. We either use all 26 electrodes, only the 3 occipital electrodes, or only the 4 frontal electrodes. For each of these EEG feature combinations, we either calculate the relative bandpower or absolute bandpower. Additionally, we optionally add additional derived features: frontal alpha asymmetry (the difference in alpha bandpower between F4 and F3), occipital alpha asymmetry (the difference in alpha bandpower between O2 and O1), occipital beta asymmetry (the difference in beta bandpower between O2 and O1), the ratio of beta bandpower over alpha bandpower at the same electrode, and the ratio of theta bandpower over alpha bandpower at the same electrode.

Decision tree implementation (LightGBM)

The decision tree implementation used for this work is LightGBM, an efficient implementation of gradient boosting decision tree open sourced by Microsoft. LightGBM focuses on growth from larger gradients and bundling mutually exclusive features. Hyperparameters for our LightGBM model are found through a grid search interface defined as the GridSearchCV class from scikit-learn. This interface allows passing of lists of hyperparameters to be searched. Hyperparameters searched for this work are min_child_samples (0, 10, 20), max_depth (2, 4, 8), num_leaves (3, 7, 15), n_estimators (5, 10, 100).

© 2020 Rajpurkar P et al. *JAMA Network Open.*
